# Camouflage Treatment of a Severe Hyperdivergent Class II Malocclusion by Posterior Interproximal Tooth Reduction and Clear Aligners: A Case Report

**DOI:** 10.1155/crid/7107818

**Published:** 2026-05-05

**Authors:** Antonino Lo Giudice, Giuseppe Palazzo

**Affiliations:** ^1^ Department of General Surgery and Medical-Surgical Specialties, University of Catania, Catania, Italy, unict.it

**Keywords:** camouflage treatment, case report, clear aligners, interproximal tooth reduction, mandibular growth

## Abstract

Orthognathic surgery is the main treatment option to improve aesthetics and function in severe skeletal discrepancy. Orthodontic camouflage could be considered a treatment option when the patient refuses surgery. However, camouflage treatment can be challenging since it requires an acceptable compromise between achieving a stable occlusion and satisfying the patient′s aesthetic expectations. This case report highlights an uncommon camouflage treatment of a severe hyperdivergent skeletal Class II malocclusion using posterior interproximal enamel reduction (IPR) and clear aligners. A 15‐year‐old female presented with a severe skeletal Class II relationship and severe facial hyperdivergency, a cusp‐to‐cusp Class II dental relationship, moderate anterior open‐bite, and maxillary constriction. Orthognathic surgery was considered the main treatment option; however, the patient refused surgical procedures. Thus, a camouflage treatment was planned with the aim to (1) improve the sagittal, vertical, and transverse occlusal discrepancies, (2) enhance smile aesthetics, avoiding deterioration of lower‐third facial aesthetics. The treatment plan included upper and lower posterior IPR and control of upper posterior intrusion, using clear aligners and Class II elastics. Posterior IPR was preliminarily calculated considering posterior Bolton′s discrepancy and inserted into the dental VTO before proceeding with the digital setup. After 26 months of nonsurgical orthodontic treatment, normal occlusion was achieved, and the smile aesthetic was improved without deteriorating the facial profile. Residual mandibular growth has significantly contributed to the correction of the malocclusion. This case suggests that the combination of clear aligners, appropriate posterior IPR (based on Bolton′s Index), and residual growth may represent a viable alternative in selected severe Class II patients refusing surgery.

## 1. Introduction

Class II malocclusion in postpubertal stage or adulthood often requires camouflage treatment due to the completion of most growth modifications [1, 2]. At this stage, orthognathic surgery is the main option for correcting skeletal anteroposterior and vertical discrepancies while enhancing smile and facial aesthetics [3]. However, the patients′ demand for orthognathic surgery is associated with high‐perceived unattractiveness, whereas acceptance rates decline markedly when patients do not perceive the aesthetic issue enough to warrant surgical intervention [4]. In addition, patients′ satisfaction with camouflage treatment is similar to that achieved with surgical treatment, [5] highlighting the influence of self‐perception in accepting treatment proposal.

Camouflage treatment requires an accurate diagnosis and a treatment plan that comprehensively includes aesthetic, occlusal, and functional factors [6]. In complex cases, the orthodontist is called to achieve a balance among these factors, customizing biomechanical strategies to reach a stable occlusion and patient′s expectations. Common treatment protocols for Class II camouflage include flaring of the incisors, distalization, extractions, and interproximal enamel reduction (IPR).

IPR is widely used in orthodontics to resolve crowding or tooth size discrepancies, and to re‐establish normal tooth morphology [7]. Although typically performed on the anterior dentition, Dr. Zachrisson and Dr. Sheridan proposed posterior IPR (in the premolar–molar region) as an important space‐management strategy, especially in individuals who require reshaping of posterior dentition [8, 9]. Posterior IPR has also been suggested in combination with clear aligners as a method for refining anteroposterior corrections when the molars are in a Class I relationship, but the canines remain in a mild Class II relationship or when the discrepancy is ≤ 2 mm [10]. However, evidence on the use of posterior IPR combined with clear aligners for treating Class II malocclusion is limited.

This article describes the camouflage treatment of a severe Class II hyperdivergent female, which was treated by integrating upper and lower posterior stripping, clear aligners, and Class II elastics. After case description, the article focuses on the integration of posterior IPR with clear aligners, the potential changes in mandibular growth direction using these appliances, and the clinical challenge of planning and performing orthodontic camouflage treatment.

## 2. Case Presentation

### 2.1. Diagnosis and Etiology

**Figure 1:**
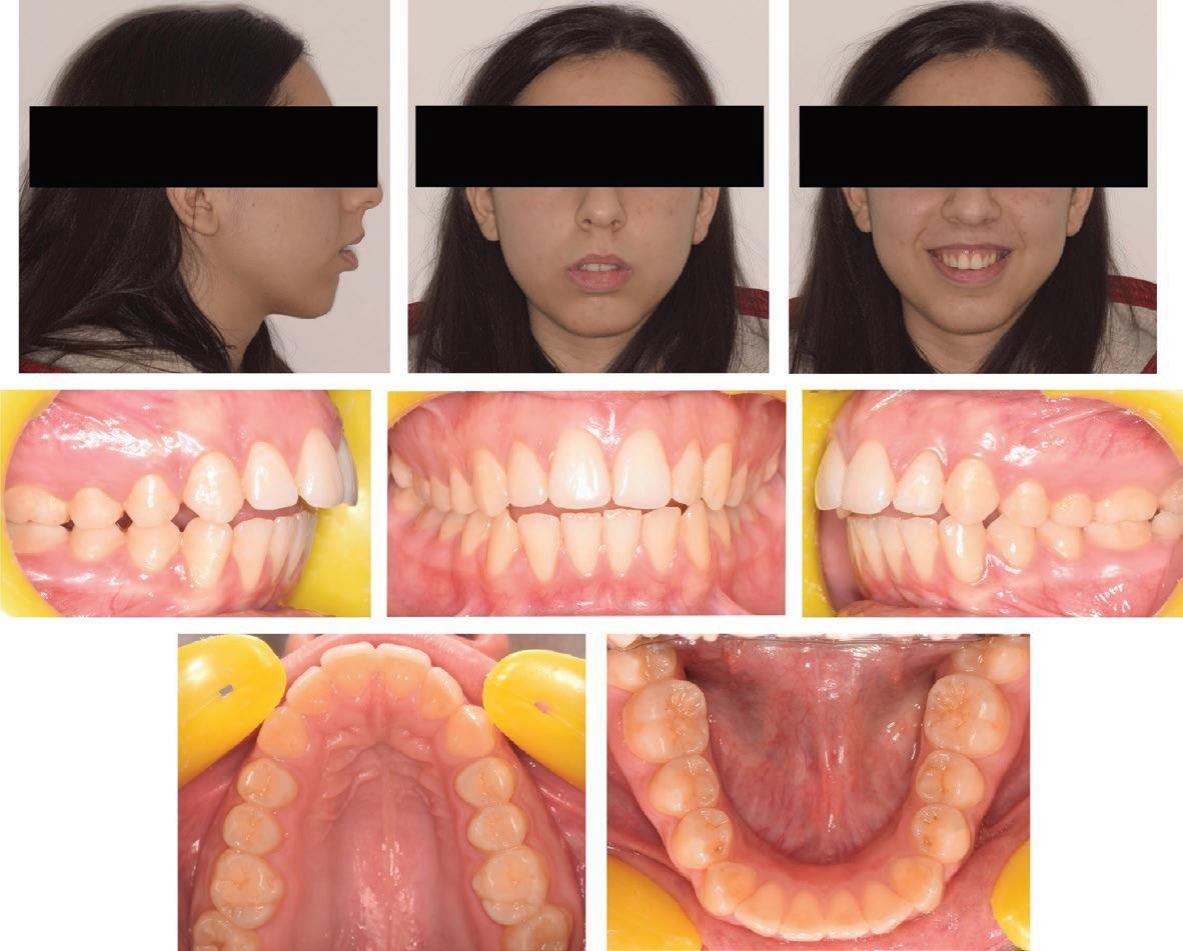
Pretreatment extraoral and intraoral photographs.

**Figure 2:**
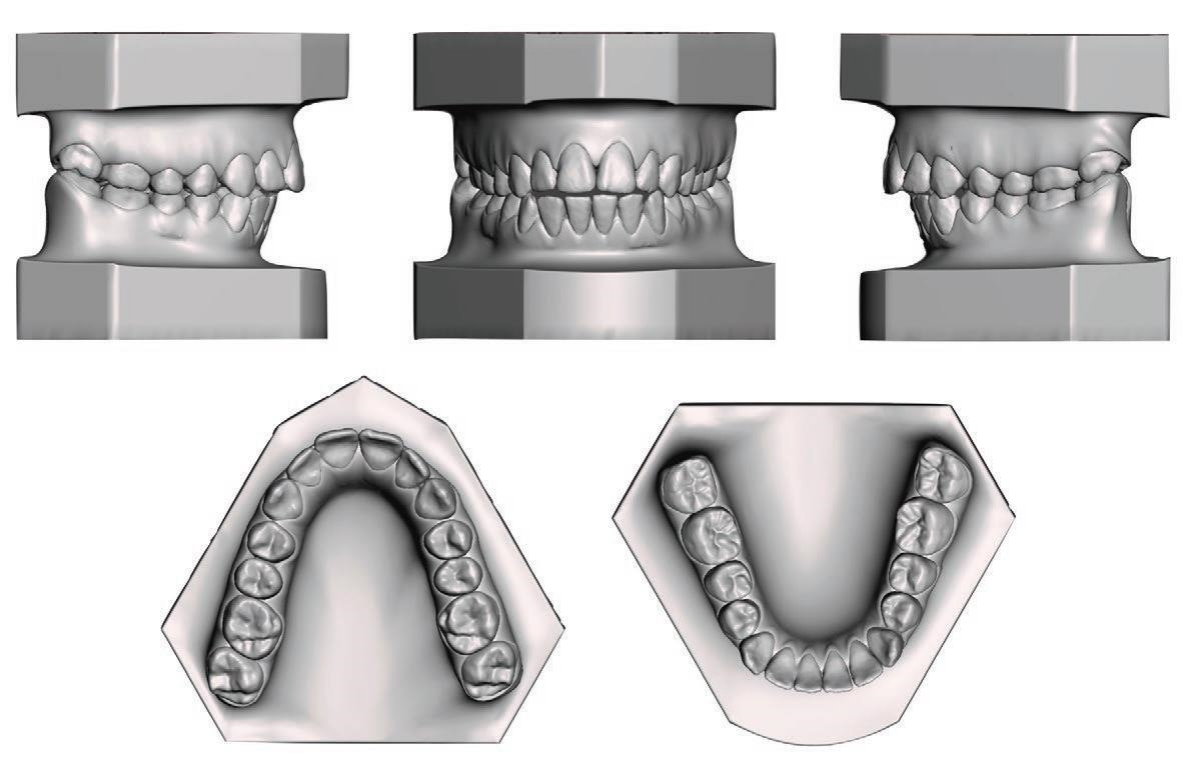
Pretreatment digital dental casts.

The present case report paper has been carried out following the CARE reporting guideline (https://www.equator-network.org/reporting-guidelines/care/). A 15‐year‐old female reported as chief complaint an excessive protrusion of the upper incisors, a narrow smile with buccal corridors and difficulty in biting food with incisors. The patient reported high concern about smile aesthetics but was not willing to undergo a surgical treatment. No relevant family history was reported. Extraoral examination showed increased facial angle with mandibular retrusion, increased lower facial height, lip incompetence and significant gingival exposure while smiling. Intraoral examination revealed a cusp‐to‐cusp molar and canine Class II relationship, 7 mm overjet, 3 mm open‐bite, 2 mm upper midline deviation (left side), maxillary contraction with V‐shaped arch and remarkable compensative lingual crown torque of the lower posterior dentition. Bolton analysis revealed a normal anterior tooth‐size discrepancy (77.24%) while the total ratio was below the ideal (86.3%), whereas mandibular premolars presented relatively rounded proximal contours and point contacts. The patient presented a thin‐scalloped gingival phenotype with poor attached gingiva (Figures 1 and 2).

The patient exhibited an anterior horizontal tongue posture and tongue thrust, which may have contributed to the open bite, the proclination of the lower incisors, and the increased curve of Spee in the maxillary arch, as described by Artese [11]. This tongue posture is consistent with a growth pattern characteristic of oral breather and long‐face syndrome. In this context, the patient reported a positive medical history of prolonged oral breathing pattern resolved after late adenoidectomy (at age 12). The patient had not received previous orthodontic treatment.

**Figure 3:**
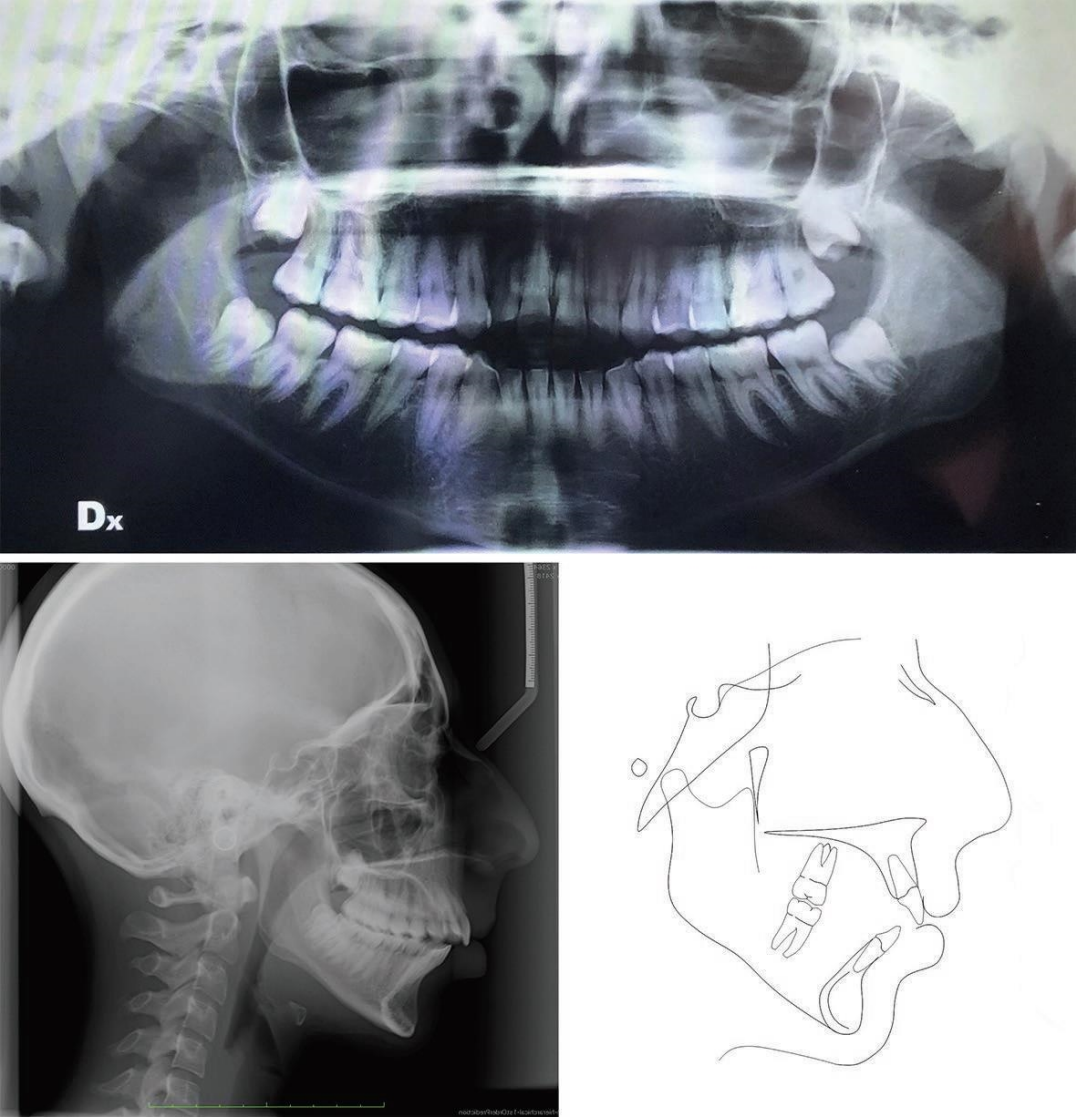
Pretreatment radiographs and cephalometric tracing.

Cephalometric analysis indicated a severe Class II relationship with remarkable mandibular divergency, clockwise mandibular rotation and lower incisor proclination (Figure 3, Table 1). The diagnosis was a severe skeletal Class II malocclusion with mandibular hyperdivergency and increased facial height, vertical maxillary excess, moderate Class II dental relationship, increased overjet, open bite and lower‐incisors proclination.

**Table 1:** Pretreatment and posttreatment cephalometric parameters.

	Normal	Pretreatment	Posttreatment	Change
*Sagittal skeletal relations*				
S‐N‐A	82° ± 3.5°	80.5°	79.9°	−0.6°
S‐N‐Pg	80° ± 3.5°	70.7°	72.2°	1.5°
A‐N‐Pg	2° ± 2.5°	9.8°	7.7°	−2.1°
*Vertical skeletal relations*				
S‐N/ANS‐PNS	8° ± 3.0°	6.7°	10.4°	3.7°
S‐N/Go‐Gn	33° ±2.5°	45.7°	44.6°	−1.1°
ANS‐PNS/Go‐Gn	25° ±6.0°	39°	34.2°	−4.8°
*Dento-basal relations*				
U1—ANS‐PNS	110° ±6.0°	108°	104°	−4°
L1—Go‐Gn	94° ±7.0°	106°	104.6°	−1.4°
L1—A‐Pg (mm)	2° ±2.0°	3.6	3.8	0.2°
*Dental relations*				
Overjet	3.5° ±2.5°	6.8°	2.8°	−4.0°
Overbite	2° ±2.5°	−1.9°	1.0°	3.0°
Interincisal angle	132° ±6.0°	109.1°	117.9°	8.8°

Abbrevitations: A‐N‐Pg, a point‐nasion‐pogonion; ANS, anterior nasal spine; Gn, gnathion; Go, gonion; L1, lower incisor; PNS, posterior nasal spine; SN, sella‐nasion; S‐N‐A, sella‐nasion‐A point; S‐N‐Pg, sella‐nasion‐pogonion; U1, upper incisor.

### 2.2. Treatment Objectives

The treatment objective was a camouflage correction of Class II by upper and lower posterior IPR and upper posterior intrusion, using clear aligners and Class II elastics. This option was proposed as a compromise orthodontic treatment to (1) improve the sagittal, vertical, and transverse occlusal discrepancies, (2) enhance smile aesthetics, (3) avoid deterioration of lower‐third facial aesthetics since surgical treatment represented the most appropriate treatment option in this patient (see treatment alternatives). The treatment plan included myofunctional therapy (the patient was referred to an expert speech therapist) to correct tongue thrust and tongue posture both during orthodontic correction and at the postretention stage. The prognosis was considered guarded due to the severe skeletal pattern and mandibular residual growth pattern.

The patient accepted the treatment objectives and strategies proposed since were consistent with her expectations, and was fully aware of the limitations in comparison to the surgical alternative.

### 2.3. Treatment Alternatives

The first option involved surgically assisted rapid maxillary expansion (SARPE) followed by Le Fort I maxillary impaction surgery with bilateral sagittal split osteotomy (BSSO) for mandibular rotation. In the case of acceptance, the orthodontic‐surgical treatment after maxillary expansion would have been deferred until the young adulthood stage. However, the patient firmly refused the orthodontic‐surgical treatment.

The second option included miniscrew‐assisted rapid maxillary expansion (MARPE) followed by orthodontic treatment supported by skeletal anchorage for posterior maxillary intrusion. This treatment option is aimed at replicating, within the limits of orthodontic movement, the directional forces of the movements planned in orthognathic surgery. In this regard, skeletal anchorage generally offers superior biomechanical control in severe hyperdivergent Class II patterns [12, 13].

However, the patient refused the application of miniscrews or any other procedure involving a surgical approach.

### 2.4. Treatment Progress

**Figure 4:**
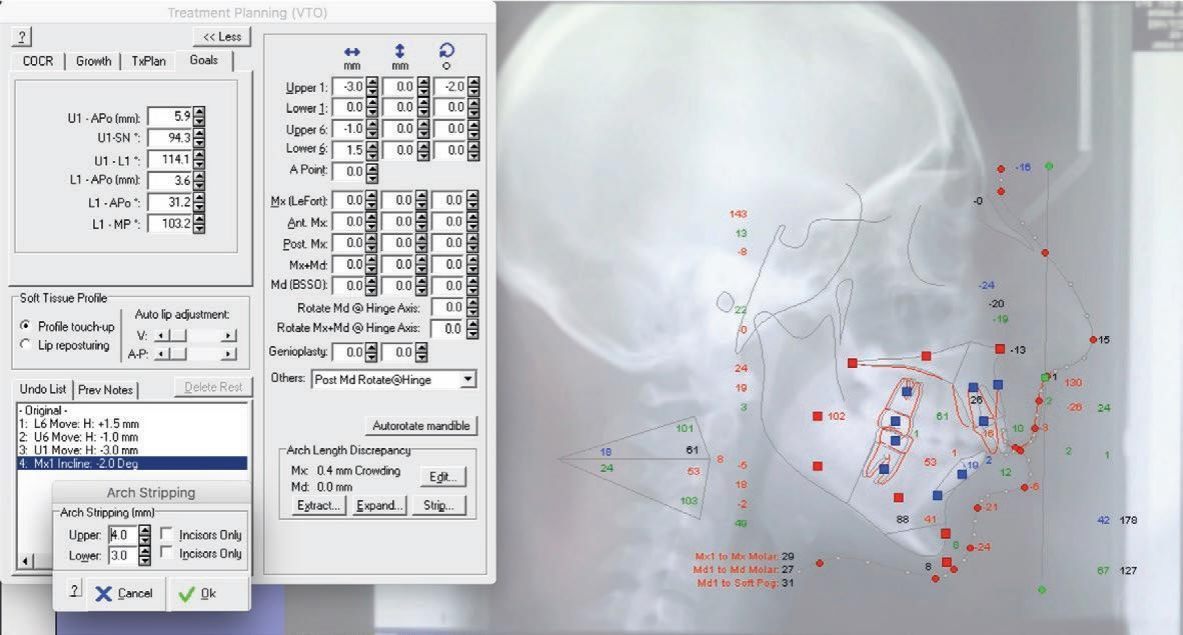
Virtual treatment objectives including posterior IPR for Class II correction. Two millimeters of IPR per side were planned in the upper arch (total 4 mm) and 1.5 mm of IPR per side were planned in the lower arch (total 3 mm) to correct a cusp‐to‐cusp molar and canine relationship. The compensatory position of the lower incisor was maintained.

A virtual treatment objectives (VTO) plan was preliminarily performed to predict the position of the incisors according to the treatment plan established, providing detailed information for the subsequent digital setup for clear aligners. Posterior IPR was planned in the upper arch (2 mm per side, total IPR = 4 mm) and in the lower arch (1.5 mm per side, total IPR = 3 mm) to correct the molar and canine relationship. The amount of IPR planned in the upper arch and lower arch was adequate to correct the cusp‐to‐cusp Class II relationship and was consistent with Bolton′s discrepancy index localized in the posterior segments (upper IPR) and with the reshaping of mandibular premolars presenting rounded proximal contours and point contacts (lower IPR). Finally, the compensatory position of the lower incisor was maintained to avoid further retraction of the upper incisors (Figure 4).

**Figure 5:**
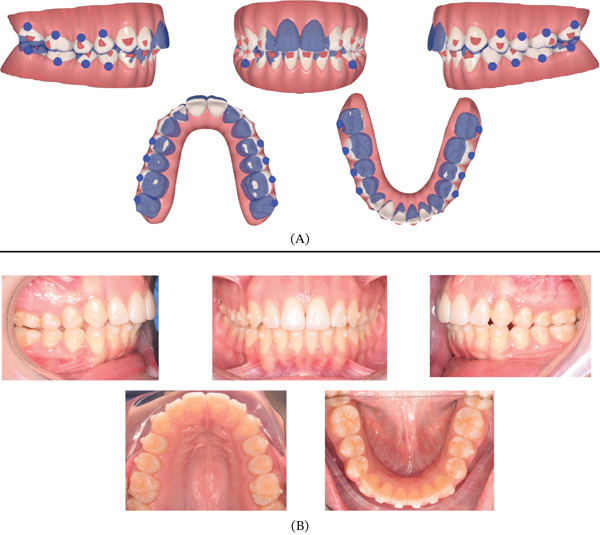
Clincheck treatment Stage 1. (A) Superimposition between pretreatment condition (blue) and planned occlusion (white); (B) occlusal results after the end of the first set of aligners.

Pretreatment impressions were sent to Invisalign to plan the treatment with clear aligners using the digital platform ClinCheck software. Two treatment stages were planned to improve the predictability of the movements. In the first stage (Figure 5A), 2 mm of posterior expansion was prescribed in association with 15° of lingual crown torque to improve arch form and the Wilson curve. In addition, 2 mm of upper posterior intrusion and 1 mm of lower incisors extrusion were required to correct the anterior openbite. Rectangular and optimized attachments were placed to improve the predictability of the movement; in particular, attachments in the upper lateral incisors served to avoid the risk of clear aligner slippering. At this stage, the patient was asked to wear each aligner for 14 days and after 8 months (17 aligners), the patient showed a significant improvement of maxillary and mandibular arch form and of the anterior openbite (Figure 5B).

**Figure 6:**
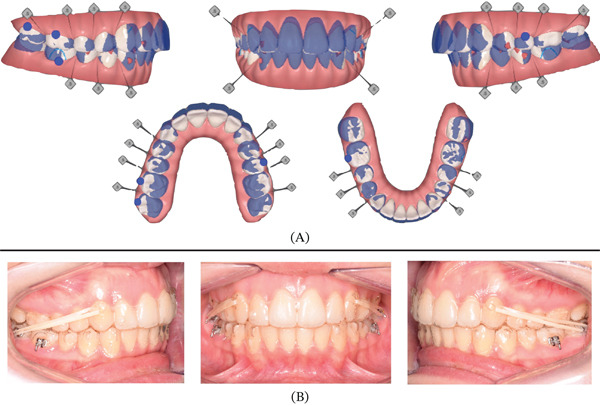
Clincheck treatment Stage 2. (A) Superimposition between the occlusion achieved after Stage 1 (blue) and final planned occlusion (white). See posterior stripping planned in both upper and lower arches. (B) Treatment progress with patient wearing Class II elastics from lower first molars (daily time) and from lower second molars (nighttime) to favor the mesialization of lower second molars.

**Figure 7:**
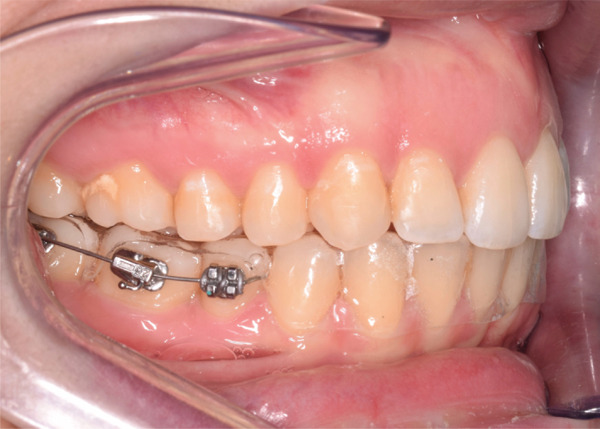
Hybrid system to correct the abrupt mesio‐inclination of the lower right molar. The retainer′s thickness was progressively reduced on the mesial half, whereas the distal half maintained the occlusal contact with the lower right molar, acting as a pivot to optimize distal tipping.

After new dental impressions, a second set of clear aligners was prescribed to correct the sagittal discrepancy. The prescription included the IPR planned via VTO, maximum anchorage on upper second molars, precision buttons on lower first molars and precision cuts on upper canines for Class II elastics, reverse curve on the upper arch to counteract the roller coaster effects consequent to space closure and incisors retraction with Class II elastics. Ten degrees of lower incisors lingual crown torque were also required to counteract the prolonged usage of Class II elastics at this stage. This stage required 20 aligners and the rate of tooth movement was set at 0.15 mm per each aligner (Figure 6A). The posterior IPR was performed progressively during the treatment stage following a clinical consolidated protocol, [8, 14] and the total amount of IPR performed per side was 2.1 mm (1.3 mm upper arch, 0.8 mm lower arch). The patient was asked to wear 4/16 Class II elastics full day and to change aligners every 10 days and was consistently monitored every 3 weeks. During treatment progress, it was noted the presence of diastema between lower second molars and lower first molars suggesting that the lower second molars did not adequately follow the lower first molars during mesialization. For this reason, a button cut was created in‐office on lower second molars for each remaining aligner and the patient was asked to wear 5/16 Class II elastics at the night‐time while maintaining the conventional configuration during the day (Figure 6B). The treatment time at this stage was 7 months. At the end of this stage, it was noted a mesio‐inclination of the lower right first molar likely due to an uncontrolled tipping under the supplementary forces produced by Class II elastics. The presence of a wide precision cut (in‐office) and the absence of attachment could explain this undesired movement. As a consequence, a hybrid appliance consisting of a sectional archwire and an essix‐type reinforced retainer was used to upright the lower right molar (3 months) (Figure 7). After correction, a final set‐up including eight aligners (aligner change = 7 days) was required for refinement. In such refinement, 5° of lingual crown torque were requested on lower incisors to improve anterior light contact. Another essix‐type retainer with precision cut for Class II elastics was worn at night for 6 months before final retention (fixed retention in the lower arch and wrapped Hawley retainer in the upper arch). Compliance was assessed throughout the treatment at each visit and was considered excellent based on aligner fit and patient reports. Table 2 describes the timeline of the present case report.

**Table 2:** Treatment timeline of the present case report.

Timeline	Description	Information
Age 15	Orthodontic consultation/case discussion	Case study, virtual treatment objectives, first Invisalign Clincheck approval
Month 0	Orthodontic treatment start	First aligners stage
Month 8	End aligners Stage 1	Dental impression—second Invisalign Clincheck approval
Month 15	End aligners Stage 2	Hybrid system for correcting lower molar mesio‐inclination
Month 18	End hybrid system	Dental impression—third Invisalign Clincheck approval
Month 20	End aligners Stage 3	Active night retainer: essix‐type retainer with precision cut for Class II elastics
Month 26	End treatment	Final records acquisition
+24 months	Follow‐up	Follow‐up records acquisition

### 2.5. Results

**Figure 8:**
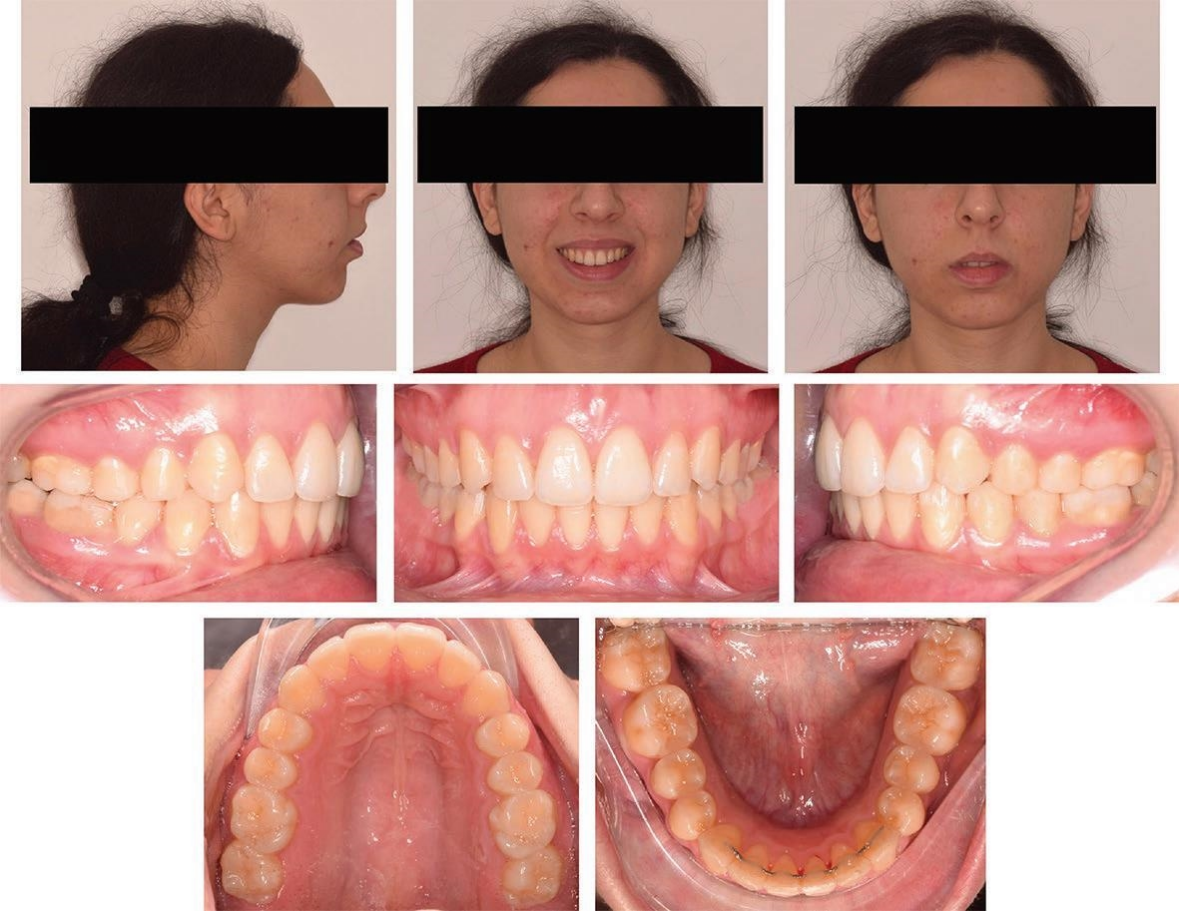
Posttreatment extraoral and intraoral photographs.

**Figure 9:**
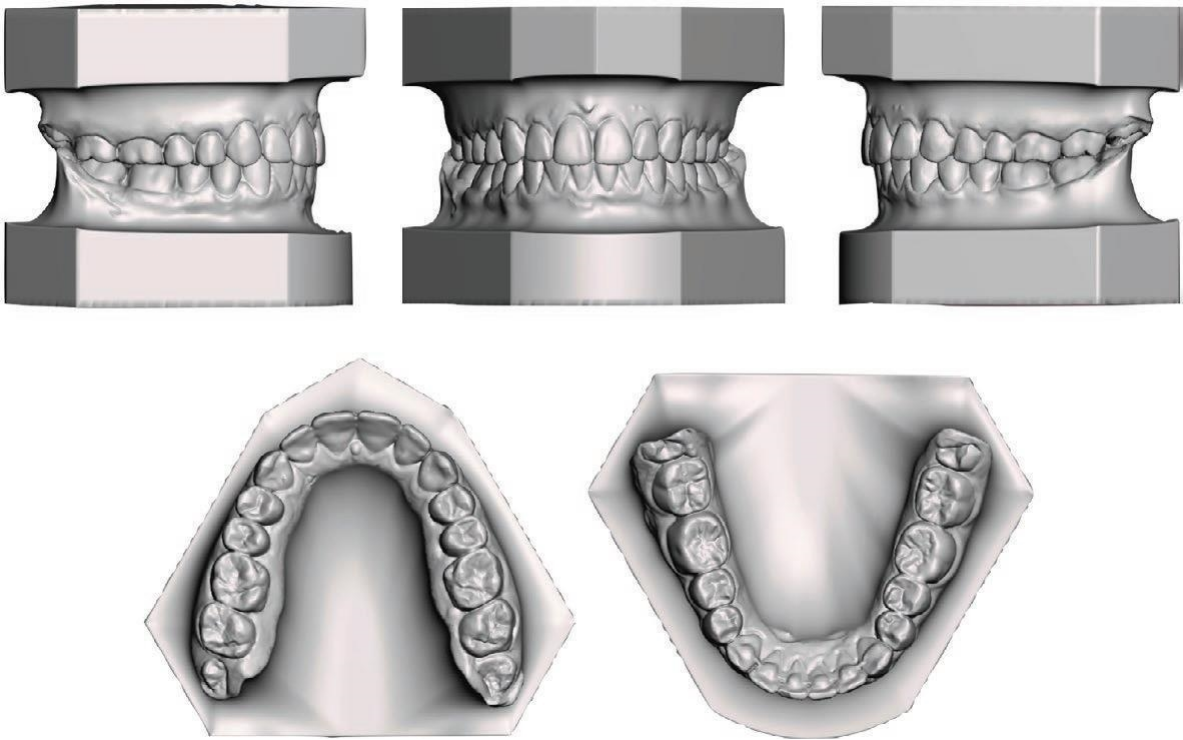
Posttreatment digital dental casts.

**Figure 10:**
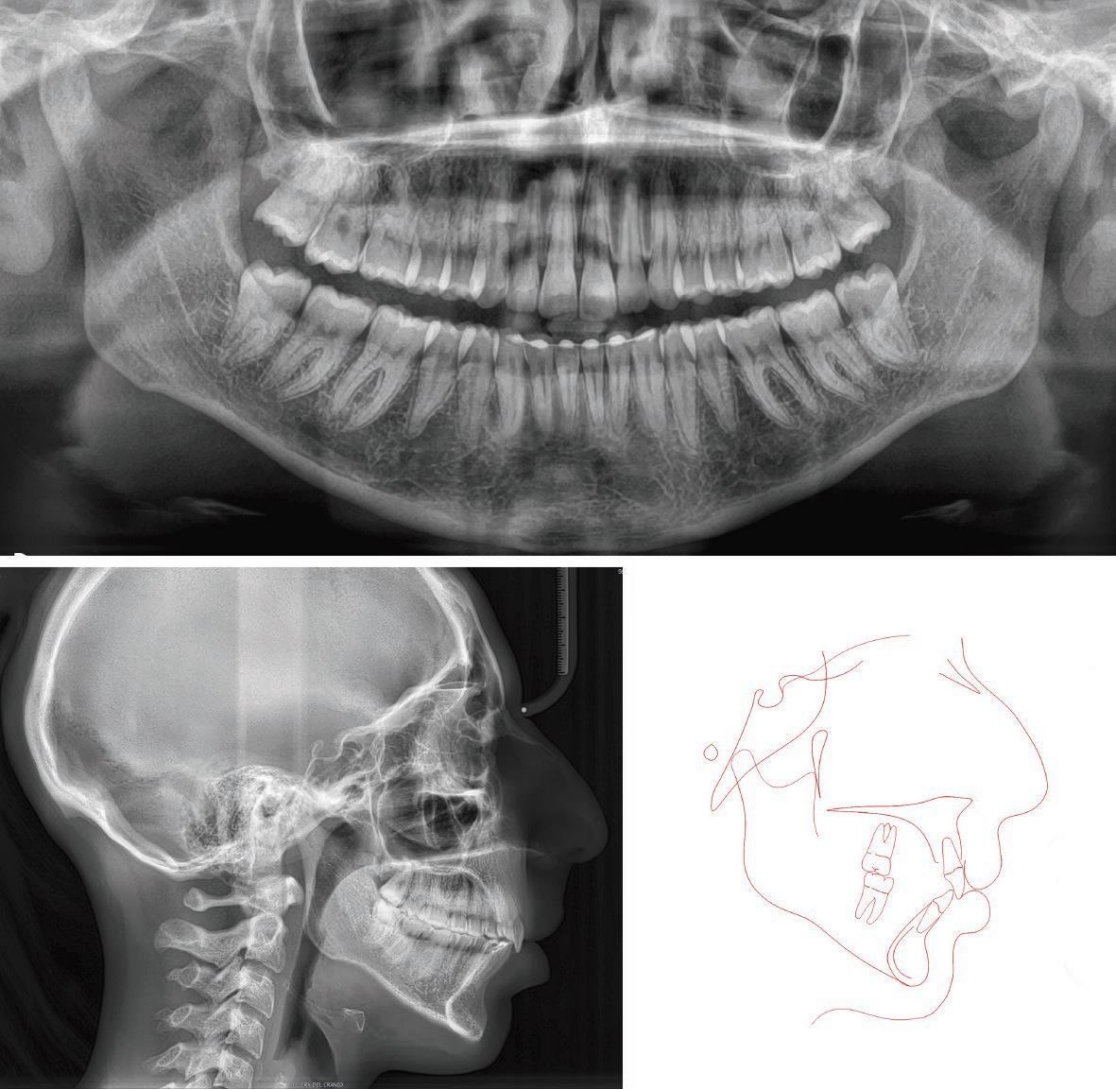
Posttreatment radiographs and cephalometric tracing.

The orthodontic treatment required 26 months to achieve the camouflage objectives planned. Class I molar and canine relationships were established, with ideal overjet and overbite, and the upper and lower midlines were coincident with the facial midline (Figures 8 and 9). The gingival tissue appeared healthy, with no gingival recession and sufficient attached gingiva. As expected, the gingival exposure and profile did not improve as these issues could only be addressed surgically. The smile aesthetics enhanced due to the absence of buccal corridors and a better consistency of the smile curve. In addition, the exposure of the lower lip vermillion was slightly reduced since impingement of the upper incisors over the lower lip was eliminated by correcting the overjet (Figure 8). The posttreatment panoramic radiograph showed well‐aligned and parallel roots (Figure 10).

**Figure 11:**
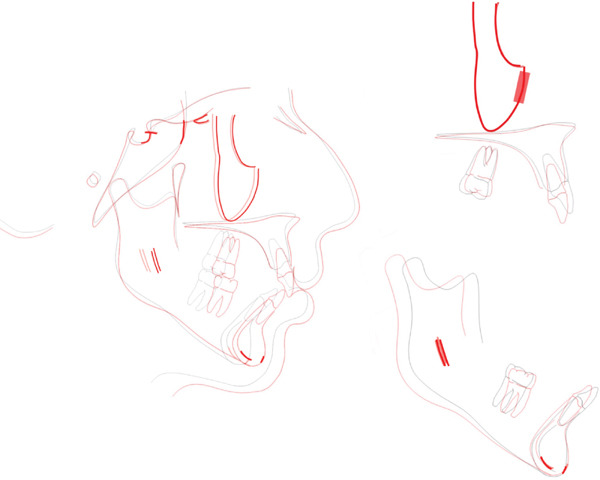
Superimposition of pretreatment (black) and posttreatment (red) cephalometric tracings.

Concerning cephalometric changes (Table 1, Figure 10), skeletal characteristics were maintained, whereas there was a significant retroclination of the upper incisors; however, this occurred within the lower limit of normal aesthetic values. [15] Superimposition showed that the dental movement planned for Class II correction was achieved, that is, maximum molar anchorage in the upper arch and molar mesialization in the lower arch (Figure 11).

**Figure 12:**
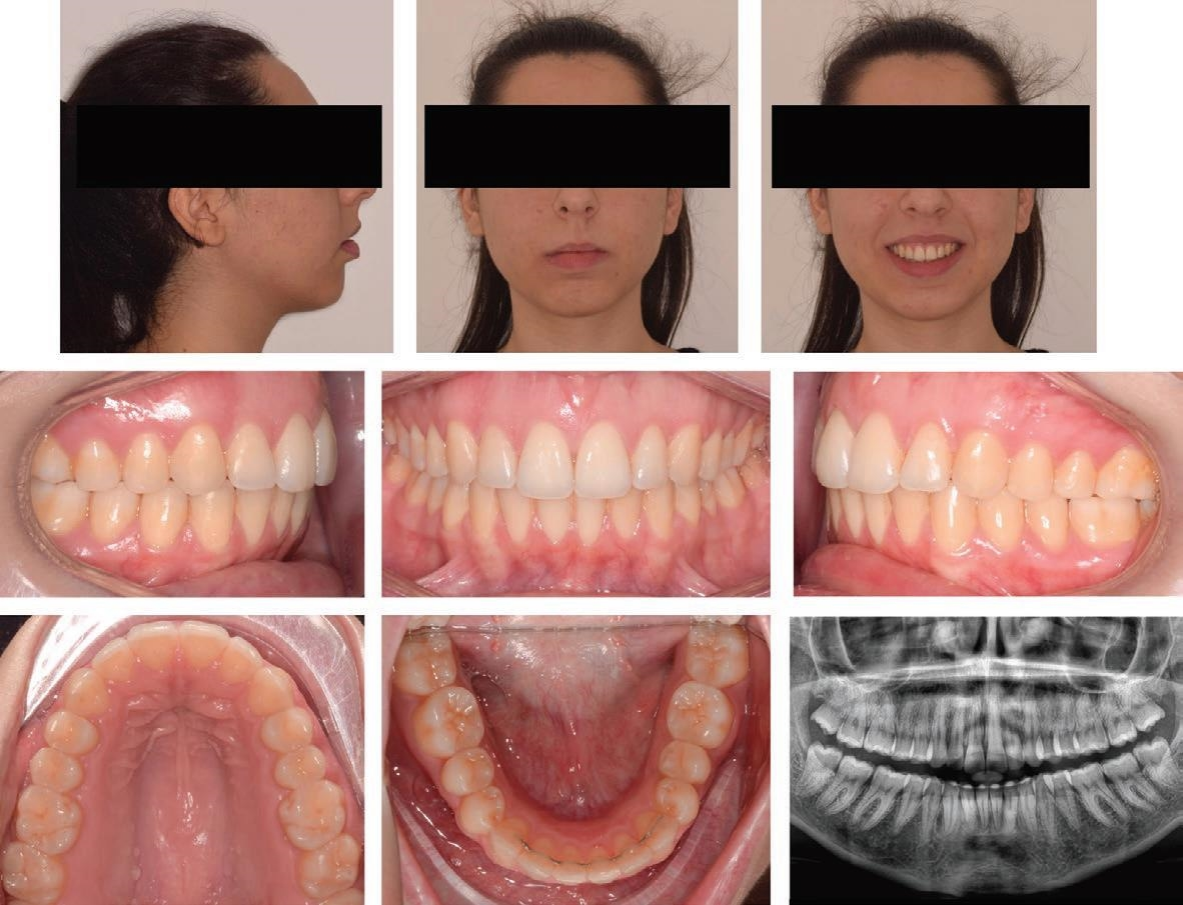
Extraoral, intraoral photographs and panorex 2 years after treatment.

Superimpositions clearly showed the main changes occurred in this patient at the end of the treatment; in particular, the combination of dentoalveolar changes (mesialization of lower molars, slight upper molar intrusion, and controlled incisor inclination) and skeletal‐positional changes (limited amount of mandibular residual growth and mandibular positional adaptation with anterotation) have contributed to the correction of the Class II. These changes would also explain why the amount of stripping required in the lower arch was less than planned on ClinCheck. Slight positional adaptation of the mandibular position may have also contributed to the long‐term stabilization of the results (Figure 12) while myofunctional therapy, by correcting the anterior tongue posture, likely has contributed to the stability of both upper and lower incisors.

**Figure 13:**
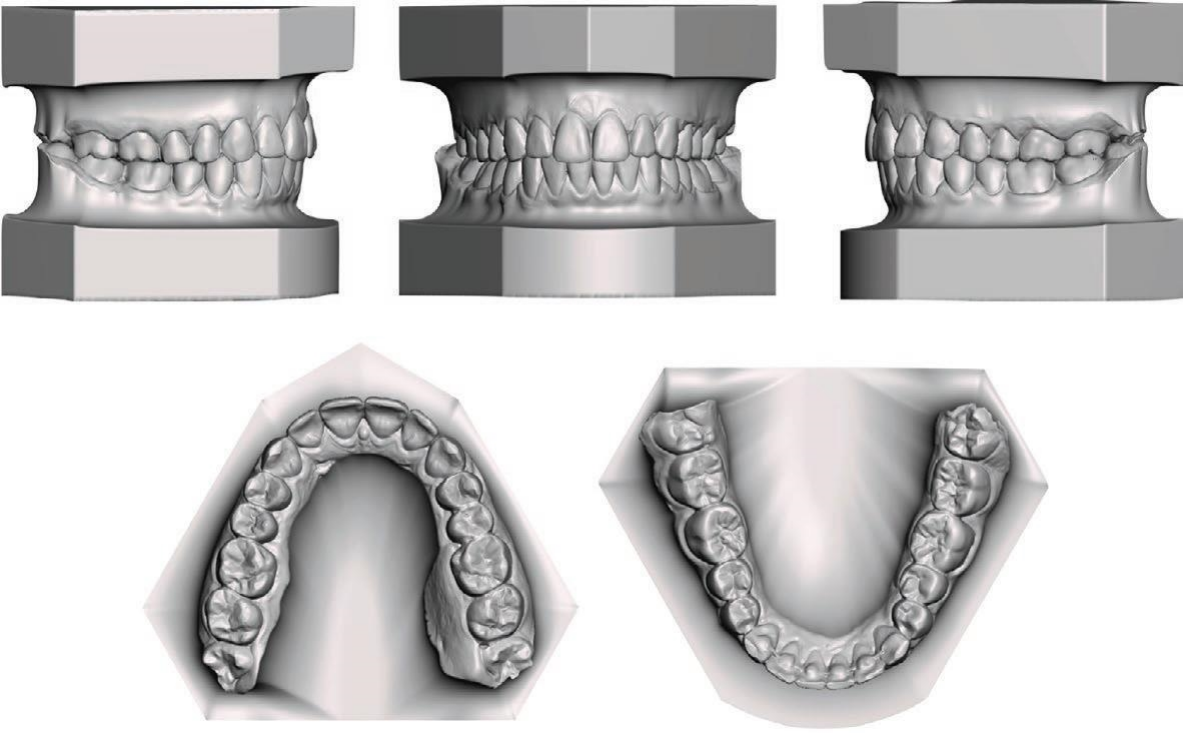
Two‐years posttreatment digital dental casts.

Patient was completely satisfied with the functional and aesthetic outcomes achieved and was scheduled for extraction of the upper third molars following the completion of orthodontic treatment. After 24 months in retention, the treatment results were stable with no clinical, radiographic, or reported signs of tooth damage (Figures 12 and 13).

## 3. Discussion

### 3.1. Treatment Plan for Camouflage Class II Correction

Undoubtedly, the treatment plan conducted represented a serious therapeutic compromise. Dentoalveolar compensation can improve occlusal relationships and smile aesthetics but cannot replace orthognathic surgery in correcting the underlying skeletal discrepancy and related aesthetic disharmonies. However, to what extent can an orthodontist accommodate a patient′s preferences differing from ideal treatment principles? This question provocatively reflects a realistic clinical scenario in which the patient refuses orthognathic surgery and the answer lies in finding the best possible compromise between the ideal treatment option and the patient′s expectations and perceptions [16].

In this patient, treatment goals were to accomplish the aesthetic expectations of the patient (reducing upper incisor proclination and buccal corridors) while primarily limiting the risk of further deteriorating facial attractiveness. Other possible camouflage treatment options could be upper arch distalization and premolar extractions. However, distalization and extractions may have deteriorated facial aesthetics considering the skeletal and soft tissue characteristics of the patient. In particular, distalization would have led to further retraction of the upper incisors, likely reducing the projection of the upper lip and resembling a Class III soft‐tissue relationship. Instead, premolar extraction would have likely improved lip competence but at the cost of flattening the profile.

The final position of the upper incisors was within the acceptable limits of smile aesthetics, that is, with the incisor′s crown parallel to the true vertical line (TVL), although they remained protruded. Smile aesthetics is known to be more sensitive to buccolingual incisors inclination than its anteroposterior position; indeed, patients and lay‐persons positively rate smile attractiveness even in presence of protruded incisors provided that the crown remain closely parallel to the TVL [17, 18]. This was confirmed by the patient′s satisfaction exhibited at the end of the treatment.

### 3.2. Digital Planning, IPR and Clear Aligners

The use of clear aligners was chosen for three potential biomechanical advantages relevant to this case: posterior vertical control without auxiliary devices, efficient space closure via IPR and control of lower incisor position.

#### 3.2.1. Posterior Vertical Control

Clear aligner therapy (CAT) is assumed to emulate occlusal bite blocks effects, limiting posterior tooth extrusion and potentially facilitating intrusion. While the literature offers limited evidence to fully support this assumption [19], in this patient a slight posterior intrusion was achieved (see superimpositions), although it remains unclear whether this effect resulted from the prescribed active movement or from the prolonged use of aligners over 26 months, including 6 months of retention with Class II elastics before the cephalometric evaluation. Furthermore, cephalometric analysis showed a reduction in the ANS‐PNS/Go‐Gn angle, which was approximately four times greater than the reduction observed in the SN/Go‐Gn angle after treatment. Therefore, the vertical changes observed are more likely related to vertical maxillary control (prevention of further posterior extrusion and dentoalveolar compensation) rather than to true molar intrusion.

#### 3.2.2. Efficient Space Closure via IPR

Clear aligners well integrate with IPR since it acts as closed systems where tooth movement is driven into the planned space by aligner‐generated pressure [10, 20]. In this patient, rectangular attachments were planned on posterior dentition to control tipping. At this stage, the rate was reduced to 0.15 mm per aligner since a movement range of 0.10–0.18 mm is recommended for patients with a thin gingival phenotype to prevent strain on the alveolar ridge [21]. In addition, the aligner change interval was shortened to 10 days to minimize the exposure to plastic degradation and ensure proper aligner fit on the teeth and attachments.

Posterior IPR was performed progressively during the treatment stage and occlusal improvement was monitored to consistently verify the necessity to reach the amount of stripping planned. Cephalometric superimposition confirmed that postpubertal mandibular growth has contributed to the correction of the skeletal Class II discrepancy and to the achievement of a proper occlusion. As a consequence, the final amount of IPR required was less than that proposed via VTO and planned on ClinCheck (2.1 = 1.3 mm upper arch, 0.8 mm lower arch), further preserving enamel thickness.

#### 3.2.3. Control of Lower Incisor Position

Planning the anchorage system a priori with clear aligners was helpful in this patient, which necessitated maximum anterior anchorage in the lower arch. Class II elastics were utilized to aid posterior mesialization by utilizing the spaces generated throughout IPR; this may have also prevented unwanted force from being transmitted from the aligner to the anterior segment, thereby allowing the expression of the planned lingual crown torque and resulting in a slight, controlled retroclination of the mandibular incisors.

An oversight in the treatment plan was the omission of attachment on the lower molars, resulting in excessive mesial tipping of the lower right first molar. This was corrected during refinement using a hybrid system—a sectional wire with an ultratipped molar tube to facilitate distal tipping, and a 1.5 mm Essix retainer serving as an anchorage unit. The retainer′s thickness was progressively reduced on the mesial half, whereas the distal half maintained the occlusal contact with Tooth 46, acting as a pivot to optimize distal tipping.

Previous studies have emphasized the importance of optimized attachment design to improve force systems and enhance root control during bodily tooth movement with aligners [22, 23]. In similar cases, the use of larger vertical rectangular attachments or optimized root‐control attachments on molars may improve the predictability of mesialization mechanics. In addition, when interproximal reduction is combined with Class II elastics, careful monitoring of molar inclination is recommended, as elastics may introduce mesial tipping moments if anchorage control is insufficient.

### 3.3. Mandibular Changes

Mandibular superimposition showed residual growth and remodeling of the mandible. This is consistent with longitudinal evidence suggesting that while absolute changes in craniofacial dimensions in Class II subjects after puberty are minimal, such limited growth (and the lack of significant growth differences compared with Class I subjects during the same period) could contribute to the improvement of orthodontic outcomes for Class II malocclusion into young adulthood [24]. However, the contribution of residual growth should be regarded as only a modest component among multiple interacting factors contributing to the sagittal improvement observed in the superimposition, that is, molar mesialization, controlled incisor inclination, and occlusal adaptation. In this regard, dentoalveolar and positional adaptations are well‐documented findings in postpubertal Class II correction using intermaxillary elastics [25]. Since the amount of such changes is not predictable at the time of the treatment plan, the correction of posterior Bolton′s discrepancy via IPR should be performed progressively and monitoring occlusal changes.

### 3.4. Posterior IPR

Zachrisson and Sheridan proposed posterior IPR as an effective finishing procedure to create space for alignment or improve occlusal settling in cases of altered Bolton ratio or over‐extended restorations [8, 9, 26]. Posterior IPR has also been suggested in combination with clear aligners and Class II elastics for correcting mild Class II discrepancies (≤ 2 mm) and sequential distalization for moderate discrepancies (≥ 2 to ≤ 4 mm) [10]. According to the JCO survey of orthodontic procedure, [27] apparently few orthodontists perform posterior IPR routinely due to concerns of causing hypersensitivity or increased risk of demineralization.

With this basis, posterior IPR in the upper arch was planned to correct the overjet and was consistent with the Bolton′s discrepancy index localized in the posterior segments; posterior IPR was also planned in the lower arch and was adequate to support Class II correction and was consistent with the reshaping of mandibular premolars presenting rounded proximal contours and point contacts. Overall, 2.1 mm of IPR was performed, corresponding to 0.3 mm per interdental space or 0.15 mm per tooth surface. This value was within the recommended range to avoid iatrogenic effects [9]. Nevertheless, the volume of enamel removed does not seem to be a significant risk factor, given that the notion that fluoride‐rich outer enamel provides protection against demineralization has been questioned [28]. Indeed, the influence of fluoride on demineralization and remineralization processes within the biofilm would be more critical than its concentration in the enamel′s apatite matrix. Regular exposure to fluoride, whether from toothpaste or mouth rinses, can help sustain this protective effect. The patient was on fluoride mouth rinse on a regular basis, and we did not perform additional in‐office fluoride treatment.

On the contrary, the key to minimizing iatrogenic complications lies in the technique used during IPR. Continuous air‐spray cooling is important to prevent odontoblast aspiration into the dentinal tubules, which signals tissue damage. In addition, steps must be avoided as they can lead to plaque accumulation and caries [14] and for this reason abrasive disks mounted on a contra‐angle handpiece are recommended for reproximation. In this patient, we followed Zachrisson′s recommendation: firstly, we applied elastics separators for 30 min, which allows a transient space to introduce the strips without pain and to improve the anatomical contours rather than just slicing. Afterward, we used in sequence a10‐mm flexible diamond disc at 20,000 rpm, 0.08 mm handheld strips for refinement and rounding corners, and Soflex disks for final polishing. The patient reported no hypersensitivity or dental damage during treatment or at the 1‐year follow‐up, and panoramic radiographs showed no carious lesions.

### 3.5. Long‐Term Assessment

Long‐term stabilization of the results (Figure 12) may have been further aided by the slight positional adaptation of the mandibular position consistent with the cephalograms′ superimposition. Furthermore, the correction of a long‐standing habit of tongue thrusting at rest and during swallowing could have also contributed to the long‐term stability of the achieved orthodontic results, in particular the position of the upper and lower incisors. In this regard, there is some evidence that orofacial myofunctional therapy can be effective in adults (although reported for different outcomes) [29], which may lead to improvements in tongue posture and orofacial function that support long‐term orthodontic stability.

Overall, the findings of the present case report cannot be generalized beyond the specific clinical conditions described. Although the treatment outcome remained stable during the available follow‐up period, longer observation is required to evaluate long‐term stability.

#### 3.5.1. Patient Perspective

The patient reported high satisfaction with both the functional and aesthetic outcomes of the treatment. She particularly appreciated the improvement in her smile and bite, as well as the possibility of avoiding surgical intervention. The use of clear aligners was perceived as comfortable and compatible with her daily activities and social life.

Although the treatment required long‐term commitment and consistent use of elastics, she found the therapy manageable and motivating due to the progressive improvement observed over time. The patient also reported increased self‐confidence following treatment and expressed satisfaction with the stability of the results during the follow‐up period.

## 4. Conclusions

Orthodontic camouflage of severe hyperdivergent Class II malocclusion represents a treatment compromise when orthognathic surgery is declined and requires careful control of biomechanical limits and treatment objectives. However, such compromises should be carefully considered within the framework of patient‐centered treatment planning and personalized medicine when patients refuse orthognathic surgery.

This case illustrates that the integration of posterior interproximal enamel reduction, clear aligners, and Class II elastics may represent a feasible camouflage option in selected Class II patients, particularly in the presence of a mild occlusal Class II relationship and posterior Bolton discrepancy, allowing improvement of sagittal occlusion and smile aesthetics.

## Author Contributions

**Conceptualization**, A.L.G.; **methodology**, A.L.G.; **writing,** A.L.G.; **clinical work**, A.L.G.; **proofreading**, G.P.; **editing**, A.L.G.; **validation**, A.L.G. and G.P.

## Funding

No funding was received for this manuscript. Open access publishing facilitated by Universita degli Studi di Catania, as part of the Wiley ‐ CRUI‐CARE agreement.

## Consent

Written and verbal consent was provided by the patient to publish this report as well as the use of clinical details, photographs, and radiographs, in accordance with the journal′s consent policy. According to institutional policies and national regulations, no additional consent or ethical committee approval was required for this single‐patient case report.

## Conflicts of Interest

The authors declare no conflicts of interest.

## Data Availability

The data of the manuscript are available upon request to the corresponding author.
